# The Contribution of Wastewater to the Transmission of Antimicrobial Resistance in the Environment: Implications of Mass Gathering Settings

**DOI:** 10.3390/tropicalmed5010033

**Published:** 2020-02-25

**Authors:** Nour Fouz, Krisna N. A. Pangesti, Muhammad Yasir, Abdulrahman L. Al-Malki, Esam I. Azhar, Grant A. Hill-Cawthorne, Moataz Abd El Ghany

**Affiliations:** 1 The Westmead Institute for Medical Research, The University of Sydney, Sydney, NSW 2145, Australia; nour.fouz@gmail.com; 2 Department of Biochemistry, Faculty of Science, King Abdulaziz University, Jeddah 21589, Saudi Arabia; alalmalki@kau.edu.sa; 3 School of Public Health, Faculty of Medicine and Health, The University of Sydney, Sydney, NSW 2006, Australia; kpan4827@uni.sydney.edu.au (K.N.A.P.); grant.hill-cawthorne@sydney.edu.au (G.A.H.-C.); 4 Special Infectious Agents Unit, King Fahd Medical Research Center, King Abdulaziz University, Jeddah 21589, Saudi Arabia; yasirphr@gmail.com (M.Y.); eazhar@kau.edu.sa (E.I.A.); 5 Department of Medical Laboratory Technology, Faculty of Applied Medical Sciences, King Abdulaziz University, Jeddah 21589, Saudi Arabia; 6 The Marie Bashir Institute of Infectious Diseases and Biosecurity, The University of Sydney, Sydney, NSW 2145, Australia; 7 The Westmead Clinical School, Faculty of Medicine and Health, The University of Sydney, Sydney, NSW 2145, Australia

**Keywords:** antimicrobial resistance (AMR), antimicrobial resistant bacteria (ARB), antimicrobial resistant genes (ARG), wastewater, Hajj and Kumbh Mela

## Abstract

Antimicrobial resistance (AMR) is the major issue posing a serious global health threat. Low- and middle-income countries are likely to be the most affected, both in terms of impact on public health and economic burden. Recent studies highlighted the role of resistance networks on the transmission of AMR organisms, with this network being driven by complex interactions between clinical (e.g., human health, animal husbandry and veterinary medicine) and other components, including environmental factors (e.g., persistence of AMR in wastewater). Many studies have highlighted the role of wastewater as a significant environmental reservoir of AMR as it represents an ideal environment for AMR bacteria (ARB) and antimicrobial resistant genes (ARGs) to persist. Although the treatment process can help in removing or reducing the ARB load, it has limited impact on ARGs. ARGs are not degradable; therefore, they can be spread among microbial communities in the environment through horizontal gene transfer, which is the main resistance mechanism in most Gram-negative bacteria. Here we analysed the recent literature to highlight the contribution of wastewater to the emergence, persistence and transmission of AMR under different settings, particularly those associated with mass gathering events (e.g., Hajj and Kumbh Mela).

## 1. Introduction

### 1.1. The Current Status of AMR as a Major Global Health Challenge

Antibiotics are one of the greatest tools of medicine. However, since the development of fluoroquinolones in early 1970, no new major groups of antibacterial drugs have been developed [1]. This paucity in development is accompanied by an increasing threat of antimicrobial resistant (AMR) organisms [1,2]. AMR is the major issue posing a threat to public health, with many reports warning of the significant risk of a post-antimicrobial era in which common infections can kill [1,3,4,5]. Recently, the World Health Organization (WHO) Global Antimicrobial Surveillance System (GLASS) reported increased levels of resistance in a number of serious and common bacterial infections in many regions of the world [6]. Currently, resistant infections result in 700,000 deaths every year, but the global resistance-associated mortality is estimated to top 10 million lives per year in 2050 [2]. The European Center for Disease Prevention and Control (ECDC) and the US Centers for Disease Control and Prevention (CDC) have reported that AMR infections resulted in 25,000 and 23,000 deaths every year in high-income countries in Europe and the USA, respectively [7]. In low- and middle-income countries, AMR infections have been responsible for the deaths of 58,000 children and 38,000 adults in India and Thailand, respectively [7]. 

### 1.2. WHO AMR Priority Pathogens List 

Recently, the WHO identified 12 bacterial species and their accompanying AMR profiles that pose the greatest threat to human health [8]. This list mainly includes Gram-negative bacteria and the most common etiologic agents associated with hospital- and/or community-acquired infections. These AMR bacteria have been divided into three categories: critical, high and medium priority, according to their impact on human health and the urgency for the development of new antimicrobial drugs to treat resistant infections. The critical category includes *Acinetobacter baumannii* (carbapenem-resistant), *Pseudomonas aeruginosa* (carbapenem-resistant) and various *Enterobacteriaceae* members, including *Klebsiella* spp., *Escherichia coli*, *Serratia* spp., and *Proteus* spp. (carbapenem-resistant and extended-spectrum ß-lactamase (ESBL)-producing), which are associated with severe and, often deadly, infections, including bloodstream infections and pneumonia. The high-priority category includes *Enterococcus faecium* (vancomycin-resistant); *Staphylococcus aureus* (methicillin-resistant, vancomycin-intermediate and resistant); *Helicobacter pylori* (clarithromycin-resistant); *Campylobacter* spp. (fluoroquinolone-resistant); *Salmonella* spp. (fluoroquinolone-resistant) and *Neisseria gonorrhoeae* (cephalosporin-resistant and fluoroquinolone-resistant), which are causative agents associated with more common infections, such as general infections, gastroenteritis and gonorrhoea. The medium-priority category includes *Streptococcus pneumoniae* (penicillin-non-susceptible), *Haemophilus influenzae* (ampicillin-resistant) and *Shigella* spp. (fluoroquinolone-resistant).

### 1.3. The Main Drivers of AMR Transmission 

AMR is driven by complex interacting factors that could be described as a resistance network [9]. This network forms links between clinical factors (e.g., human health, animal husbandry and veterinary medicine) and other components, including human activities (e.g., travel [10,11], human displacement and over and misuse of antimicrobial drugs [12,13,14]) and environmental factors (e.g., persistence of antimicrobial drugs and AMR organisms in soil and water). For example, the variations in AMR patterns among different regions of the world have been associated with differing rates of consumption of, and exposure to, antimicrobial drugs [2]. This is alarming, with the data available on AMR transmission suggesting increasing consumption of antibiotics in humans during the past two decades, primarily in low- and middle-income countries [15]. The selective pressure associated with the exposure to antimicrobials in healthcare, agriculture and the environment enhances the development of new AMR variants and novel resistance mechanisms [16]. Other factors, including lack of access to clean water sanitation and healthcare service, poor personal hygiene, failure of AMR detection and treatment and poor vaccination coverage [17] in the community, have been shown to also contribute to the global transmission of AMR [18]. 

### 1.4. The Environmental Reservoir of AMR from Water and Sewage 

Transmission of AMR can spread between people, animals and the environment via a number of different routes [19]. The environment acts as a bridge for different compartments, between animals to compost to soil to water to sediments to sewage [20]. While the environment acts as the reservoir, it also works simultaneously to mix mobile genetic elements (MGEs) that interact and diffuse into other parts or into human and animal hosts [19,21,22].

Many studies have highlighted the impact of the diverse nature of the reservoirs of AMR genes (ARGs) on promoting the emergence and transmission of AMR organisms [23]. AMR is ancient and ubiquitous in the environment, with many lines of evidence suggesting that transfer of ARGs occurs among different environments (e.g., from environmental to pathogenic bacteria) [24,25]. Although it has been well-established that the genetic transfer of ARGs is likely to occur between closely-related species, recent studies have suggested that this transfer can also occur among phylogenetically distant species and even among organisms belonging to distinct phyla [26], adding further challenges in the continuous evolution of new variants of AMR organisms. High concentrations of antibiotic residues, ARGs and AMR organisms have been reported from environmental samples recovered from hospital and urban and treated wastewaters and soils treated with animal manure [27,28,29]. 

Many studies have highlighted the role of sewage as a major environmental reservoir of AMR, as it represents an ideal environment for AMR microorganisms and ARGs to persist [30,31,32]. The situation of ARGs is more complex, because they are not degradable and can be spread among microbial communities in the environment through horizontal gene transfer, which is the main resistance mechanism in *Enterobacteriaceae* [33,34].

In this study, we aimed to systematically review the literature to identify the role of wastewater in promoting the transmission of AMR and to characterise the key factors implicated in the persistence of ARB and ARGs in this environmental component. We extended the analysis to characterise AMR transmission in environmental samples associated with key religious mass gathering events—Kumbh Mela and Hajj in India and Saudi Arabia, respectively.

## 2. Materials and Methods 

### 2.1. Search Strategy 

Searches were systematically carried out in four databases: Embase, Medline, PubMed and Web of Science Core Collection to obtain all articles that reported AMR in sewage samples. The key terms “antimicrobial resistance” OR “AMR” in combination with “sewage” were used to obtain the articles available between 2009–2019, with the search conducted on 21st June 2019. EndNote X7.5 (Thomson Reuters) was used for bibliography management. The duplicates were removed, and initial screening was performed by assessing titles, abstracts and keywords with an explicit focus on the use of molecular approaches, including whole genome sequencing and metagenomics, in detecting AMR. The search was extended to include special settings, such as mass gatherings at Hajj and Kumbh Mela. 

### 2.2. Selection Criteria 

**Figure 1:**
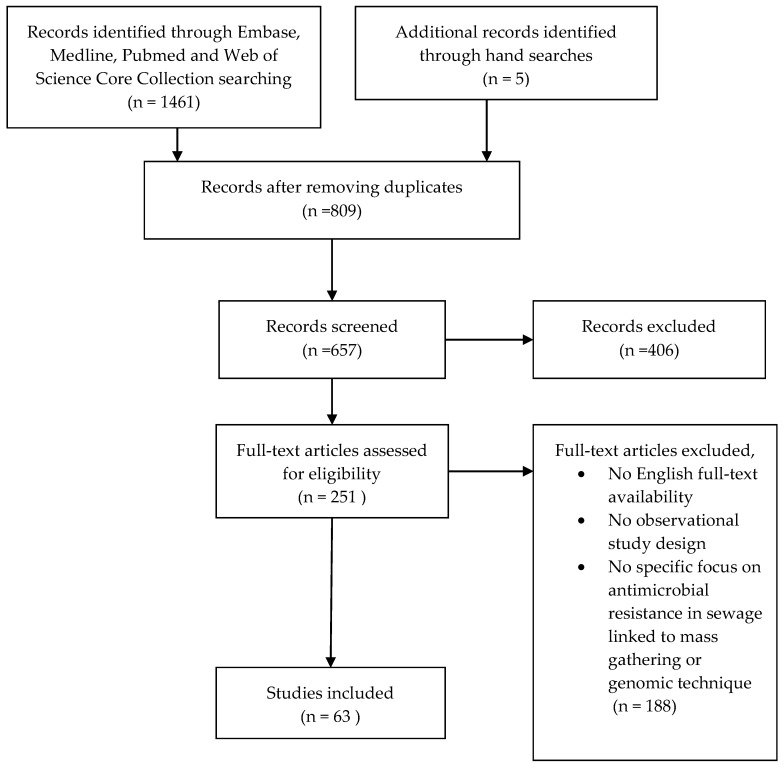
Flow-chart of literature search.

Articles were included if they were written in English and included an observational study design where sewage samples were investigated molecularly for the detection of AMR. We excluded articles if they were written in languages other than English, reviews, opinion articles and editorials. Potential articles were evaluated on the inclusion criteria by retrieving the full text and were subsequently included in the analysis (Figure 1).

### 2.3. Data Analysis 

1461 articles were obtained in the initial literature search and five articles found by hand searches, which included 809 duplicates. After removing the duplicates, the first screening removed non-English records and irrelevant abstracts, resulting in 251 remaining articles. Full-text was retrieved to screen the articles on the selection criteria, and a total of 63 papers were eligible for inclusion in the analysis (Figure 1). All papers were dissected to summarise the key information and findings, including year of publication, country study site, source and type of wastewater, abundance of ARGs and AMR microbial communities and methods used for AMR detection.

## 3. Results

### 3.1. Dissemination of Antimicrobial Resistance in Wastewater 

From the 1466 articles that were identified, 63 studies conducted on wastewater samples between 2004 and 2018 (published in the period 2009–2019) were included in the data analysis. The analysed studies documenting the detection of ARBs and/or ARGs in different types of wastewaters are listed in Table 1. The source and type of wastewater samples investigated and the key findings highlighted by these studies are summarised (Table 1). Detailed information on location and time of sample collection, structure of ARBs populations and/or ARGs detected and the technology used in AMR characterisation are provided in Table S1. 

These studies highlight the role of aquatic ecosystems, particularly wastewater, as a key reservoir of AMR bacteria and ARGs in the environment. High levels of both ARBs and/or ARGs were detected in samples collected from different types of wastewater, including municipal sewage [39,46,47,54,81] and influent and effluent samples from wastewater treatment plants (WWTPs) [37,42,44,55,63,64,70,71,72,78,79]. Similarly, high levels of AMR were identified in industrial [66,77,92] and agricultural wastewater and samples recovered from pharmaceutical treatment plants [49,66]. High levels of clinically relevant ARBs and/or ARGs were identified in influent and effluent samples from hospital wastewater [36,39,40,42,50,54,58,76,80,82,83,90,94,97]. 

A number of studies have also demonstrated elevated levels of AMR detected in samples that have been collected from downstream water [36,44]; the surface water of rivers [35,37,53,75,86,88] and tap water [56]. Few studies have detected ARBs and clinically relevant ARGs in environmental samples that have been exposed to/contaminated with sewage [62,91].

### 3.2. ARB Populations Associated with Wastewaters

The majority of the studies have used integrated molecular and phenotypic approaches to characterise the resistance profiles and virulence contents associated with AMR bacteria. Many studies (n = 47) have used advanced molecular approaches, including polymerase chain reaction (PCR) followed by Sanger sequencing and/or quantitative PCR, to characterise the AMR genotypes. Recent studies (n = 16) have used whole-genome sequencing and metagenomic analyses to comprehensively detect the microbial and AMR determinants in wastewater samples. The latter aimed to assess the abundance and distribution of microbes and associated AMR agents (mobile genetic elements (MGE), including plasmids, transposons, integrons and insertion sequences) and to identify the factors that determine the persistence of AMR bacteria and ARGs in wastewater.

Phenotypic characterisation demonstrated that *Enterobacteriaceae* members, including *Escherichia coli*, *Klebsiella* spp., *Shigella* spp., *Salmonella* spp., *Vibrio* spp., *Acinetobacter* spp. and *Enterococcus* spp., were among the most common AMR bacteria identified in the wastewater samples investigated in the analysed studies (Table 1). Additionally, high levels of MDR bacteria and ARGs conferring resistance to varied classes of antimicrobial drugs, including beta-lactams, carbapenems, tetracyclines, aminoglycosides, fluoroquinolones, sulphonamides, macrolides, vancomycin and erythromycin, were documented in the analysed articles (Table 1).

### 3.3. Selective Pressure within Wastewater Environments Promote the Emergence of Novel Variants of ARGs and ARBs

Generally, high levels of ARB, including MDR strains and diverse ARGs, have been detected in influent wastewater (untreated) collected from various sources, particularly low-income settings [41,44]; hospitals [36,39,40,42,46,47] and pharmaceutical waste [49,60]. However, many studies demonstrated that effluent samples collected from urban, hospital and pharmaceutical-treated wastewater still contain elevated levels of diverse ARGs, ARB and antimicrobial drugs [49,58,60,63]. For instance, a recent study demonstrated that the abundance of ARGs was significantly higher in effluent wastewater samples collected from low-income compared to high-income countries [41,44]. High rates of ARGs have been identified in pharmaceutical wastewater treatment plants, with the rate of those associated with clinically important antimicrobial drugs (e.g., *sul1*, *sul2* and *tet*) being found to remain high throughout the different stages of the treatment process and, therefore, were subsequently discharged into the environment [49]. 

NDM-1 producing strains, including *V. cholerae*, *Shigella boydii* and *Aeromonas caviae*, which had not been previously reported to carry *bla*_NDM-1_, have been isolated for the first time from drinking water (4%; 2 out of 50) and seepage samples (17%; 12 out of 171) from New Delhi [56]. This is in addition to the previously reported NDM-1-producing species, including *E. coli* and *K. pneumoniae* [56]. The carriages of *bla*_NDM-1-_bearing plasmids by enterobacteria, aeromonads and *V. cholerae* have been shown to be stable, transmissible and exhibit the typical resistance pattern of NDM-1 [56]. Although the majority of strains have previously carried *bla*_NDM-1_ on plasmids, *bla*_NDM-1-_bearing chromosomes have been first identified in environmental isolates of *Aeromonas caviae* and *V. cholerae* [56]. Another study has documented the isolation of novel species of *Acinetobacter cumulans* from hospital wastewater [36]. These strains have been found to contain ARGs associated with resistance to clinically important drugs, including carbapenems, cephalosporines and aminoglycoside [36]. Additionally, a carbapenemase-producing *K. pneumoniae* strain carrying *bla*_KPC-2_, which is rarely detected in clinical settings, has been identified in WWTP effluent wastewater in Japan [38].

Additionally, many studies have detected high levels of integrons [77,78,79,81], including novel classes associated with oxacillinase gene cassette (*bla*_OXA-109_, *bla*_OXA-368_ and *bla*_OXA-2_) in varied bacterial species recovered from wastewater samples [90]. Higher prevalence of class 1 integrons was detected in bacteria recovered from sewage sludge and pig slurry (environments that contain high concentrations of antibiotic residues and detergents) compared to agricultural soils to which these waste products are amended [77]. It has been estimated that ~10^19^ bacteria carrying class 1 integrons enter the United Kingdom’s environment by the disposal of sewage sludge each year [77]. In another study, the investigated β–lactamase genes (*bla*_TEM_ and *bla*_CTX-M9_) and *mecA* encoding for penicillin-binding protein were detected in all DNA phages that have been recovered from urban sewage and river water samples [75].

Collectively, the analysed studies demonstrate the potential role of wastewater (particularly untreated-, hospital- and pharmaceutical wastewaters) as an environmental reservoir that assists in the emergence and dissemination of novel variants of AMR bacteria. This is mainly promoted through the coexistence of diverse species of bacteria and high levels of ARGs in these environments, which increases the probability of the transfer of ARGs carried on mobile elements among closely related species. The untreated wastewaters also contain high levels of antimicrobial drugs, which pose an important selective pressure for the emergence and dissemination of AMR bacteria [98]. Recently, positive correlations were observed between the occurrence of heavy metals (e.g., zinc and lead and *ereB*, *mefA*&*E* and *ermB*) and antibacterial residues (e.g., triclosan with *ereA*, *ereB*, *mefA*&*E* and *ermB*) in urban wastewaters and the presence of erythromycin resistant genes [87]. However, the dynamic of the selective pressure and the emergence of novel variants of ARBs remain poorly documented and understood.

### 3.4. Hospital Wastewater and the Dissemination of Clinically Relevant ARGs and ARBs Populations 

Recent studies have reported the detection of elevated levels of clinically important AMR bacteria and ARGs in hospital effluent wastewater and environmental water sources that receive untreated hospital waste [39,46,47,50,58]. Clinically important AMR bacteria, including MDR (e.g., carbapenemase-producing *Enterobacteriaceae*) and ESBL-producing bacteria (e.g., ESBL-producing *K. pneumoniae*) [39,50,58] and vancomycin and ampicillin-resistant *Enterococcus* spp., have been identified in hospital wastewater-associated samples [46,47]. 

### 3.5. Impact of Wastewater Treatment Processes on AMR Dissemination

Conventional and advanced WWTPs have employed different biological, physical and chemical process to clean wastewater from pollutants and contaminants so that they can be reused and/or returned back to the environment. The efficiency of removal of AMR bacteria from effluent wastewater (treated) varies according to the treatment procedure employed [99]. Therefore, it is not surprising that high levels of clinically important AMR bacteria, including MDR and ESBL-producing strains of *K. pneumoniae*, *Enterobacter cloacae* and *E. coli* [58]*;* MDR *Listeria* spp. [71] and MDR *Vibrio* spp. [72], have been detected in effluent samples. Another study demonstrated the detection of MDR *E. coli* strains associated with neonatal meningitis, intestinal and extraintestinal serotypes in final effluents of WWTPs [52]. High levels of resistance were identified in BFG bacteria isolated from WWTPs compared to those that have been recovered from human faeces investigated [40].

Importantly, there is growing evidence that wastewater treatment does not have a profound impact on eliminating the ARGs present in hospital wastewater, with no significant difference in ARG abundance between influent and effluent hospital wastewater samples [54]. Another study demonstrated the high abundance of ARGs and MGEs, including plasmids, transposons, integrons and insertion sequences among samples collected during different treatment processes using aerobic activation (aerobic-activated sludge (AAS)) or anaerobic digestion (anaerobically digested sludge (ADS)) [48]. However, a distinct microbial population has been identified in AAS compared to ADS samples, which suggests a role for the treatment process in promoting the dissemination of particular resistance patterns [48]. A number of recent studies have also demonstrated that novel ARG-bearing plasmids and ARGs that confer resistance to multiple clinically relevant antimicrobial drugs, including aminoglycoside and β-lactams, were highly enriched in activated sludge and effluent wastewater [57,59,61,89,92]. Additionally, activated sludge investigated in one study was found to contain varied ARBs, including ESBL-*Enterobacteriaceae*, MRSA and VRE, and several ARGs associated with resistance to β-lactam, vancomycin (*vanA*) and methicilin (*mecA*) [95]. 

Consistently, Gram-negative and -positive isolates dominated in WWTP influent and effluent samples, respectively, with the frequency of detection of tetracycline-, sulphonamide-, streptomycin- and β-lactam-resistance genes (except *sulA* and *bla*_CTX-M_) being higher in ARB from influent compared to effluent samples [89]. The abundance of *intI1*-bearing bacteria (including *E. coli*, *Klebsiella* spp. and *Aeromonas veronii*) were higher in effluent compared to influent wastewater, with *intI1* being detected in 20.4%, 30.9% and 38.9% of bacteria recovered from influent, activated sludge and effluent wastewater, respectively [78]. In another study, MDR *Enterobacteriaceae* strains carrying class 1 and class 2 integrons (12.1%; 221 out of 1832) were identified in different stages of a municipal wastewater treatment plant (61.5%, 12.7% and 25.8% of ARB originated from raw sewage, aeration tank and final effluent, respectively) [81]. However, the abundance of ARGs and MDR bacteria, particularly the levels of ARG diversity and β-lactamase-producers, were higher in the final effluent samples [81].

Collectively, these studies demonstrated WWTPs as hotspots for the emergence of ARBs and highlighted the impact of the treatment technology employed and potential roles of specific stages of treatment processes, particularly those characterised by high biomass and biodiversity (e.g., activated sludge), in maintaining diverse ARGs and promoting particular populations of ARBs. Advanced treatment processes, including membrane filtration, ozonation and UV-irradiation, are highly efficient in reducing the abundance of AMR in effluent wastewater to levels observed in low-impacted surface water [99]. 

### 3.6. AMR Dissemination in Wastewater Associated with Mass Gathering Settings 

Most of the studies that reported AMR in wastewater have been conducted within one or a few countries. The majority of the studies were conducted in Asia (n = 22), followed by Europe (n = 23), Africa (n = 10), South America (n = 9), North America (n = 7), Central America (n = 1) and Oceania (n = 2). 

No studies have been conducted to investigate the transmission of AMR bacteria and ARGs in environmental samples associated with key religious mass gatherings (Kumbh Mela and Hajj) occurring in low-income settings. Kumbh Mela and Hajj are the largest and most diverse mass gathering events that have been associated with an increased risk of infectious disease emergence and transmission [100,101]. 

Kumbh Mela, the world’s largest religious gathering that attracts millions of Hindu pilgrims, is celebrated at four riverbank pilgrimage sites, including Ganges-Yamuna-mythical Saraswati rivers confluence, Ganges, Godavari and Shipra [101]. The bathing of the pilgrims in these rivers is one of the key rituals, as they believe that it cleanses them of their sins. This raises serious public health issues with regards to the dissemination of waterborne diseases in a setting known to be endemic for cholera [102,103]. Recently a number of studies using metagenomic approaches have detected high levels of ARB, ARGs and antimicrobial residues in water and sediment samples collected from the Ganges River [104]. In addition, ARGs related to different classes of clinically relevant antimicrobial drugs, including ß–lactams, aminoglycosides, fluoroquinolones, macrolides-lincosamide-streptogramins (MLS), rifampicin and sulphonamides, have been identified in samples collected from the confluence of the river Ganges with Yamuna [105]. 

Hajj has already been associated with an increased risk of airborne, foodborne and zoonotic infections [100]. Recent studies have demonstrated that pilgrims are at high potential risk of acquiring and transmitting AMR enteric bacteria, [106,107,108,109] including carbapenemase-producing *E. coli* [110] and extended-spectrum cephalosporin- and colistin-resistant non-typhoidal *Salmonella* [111], as well as MDR *Acinetobacter* spp. [110]. 

## 4. Discussion

The release of antimicrobial drugs, ARBs and ARGs originating from human and animal waste to the environment is a global problem that has serious implications on public health. Therefore, strengthening knowledge on the spread of AMR through surveillance and research was one of the key strategic objectives of the WHO global action plan that was launched in 2015 [112]. Here, we systematically analysed the recent literature to highlight the contribution of different types of wastewaters from various sources (e.g., low- and high-income countries and mass gathering settings) to the emergence, persistence and transmission of AMR in environments and their potential impacts on public health. 

The analysed studies highlighted the role of wastewaters as major sources of antimicrobial agents, ARBs and ARGs in the environment. Particular types of wastewaters (e.g., untreated municipal-, hospital- and pharmaceutical wastewaters) have been characterised by high levels of clinically relevant ARBs and ARGs. These environments can provide an ideal platform allowing the transfer of ARGs among the bacterial populations either before or after being discharged into the environment. This is alarming considering that many clinically relevant bacterial species, including enterotoxigenic *E. coli* and typhoidal and non-typhoidal serotypes of *Salmonella,* have been shown to be able to persist in the environmental water for relatively long times [113,114,115]. 

Wastewater treatments have been shown to be effective in reducing the ARB loads in effluent samples. However, there is increasing evidence that effluent samples from wastewater treatment plants, wastewater discharges of pharmaceutical production facilities, hospitals and other healthcare facilities are hotspots (ideal platforms) for selective pressure processes that promote the emergence and dissemination of novel AMR mechanisms and new variants of ARBs and ARGs. However, it is noted that the positive selection process and the dynamics of the emergence of novel variants of ARGs and ARBs within WWTPs and their associated impacts on human health remain poorly documented and characterised. The WHO has highlighted the need for greater attention and action to develop quantitative microbial risk assessments and supporting guidance to address human health risks associated with environmental exposures to antimicrobial agents, their metabolites, ARBs and ARGs [32]. 

Interestingly, a recent pioneer study has proposed a culture-independent metagenomic analysis of untreated wastewater as an effective approach to track and predict the dissemination of AMR bacteria and ARGs globally [41]. The authors of this study have used a standardised metagenomic protocol to characterise the bacterial resistome content and to detect variations in the abundance and diversity of ARBs and ARGs in a global collection of untreated wastewater samples (collected from 79 sites in 60 countries). This study demonstrated that clinically relevant ARGs were more abundant in samples collected from low- and middle-income settings in Africa, Asia and South America, compared to those that have been collected from high-income settings in Europe, North America and Oceania. The variations in AMR gene abundance were found to strongly correlate with socioeconomic, health and environmental factors [41]. 

This approach can be applied in challenging settings (e.g., such as low-income countries and complicated mass gathering settings) to study the paradigm of AMR dynamics and epidemiology and inform on the processes leading to the emergence and the dissemination of AMR infectious agents and, therefore, help in developing management strategies.

We conducted a research study that uses the opportunity presented each year by the Hajj pilgrimage and advanced shotgun-based metagenomic approaches to characterise the global population of enteric microorganisms circulating in environmental Hajj settings. This will provide an annual snapshot of the AMR bacteria and MGEs associated with each global locality and help in identifying the dynamics of emergence and dissemination of AMR in the environment. 

Hajj is a unique mass gathering event that has been associated with an increased potential for the emergence and dissemination of AMR infections, raising major public health concerns within the host country and globally. The enormously diverse population of 3 million pilgrims, originating from 190 countries all over the world come together to perform the same activities within a relatively short period of time and over a limited area of land. Importantly, the pilgrims are required to stay in tents in Mina (a nonpopulated valley covering approximately 20 km^2^ of land, of which only 4 km^2^ can be occupied by pilgrims) for at least 3–5 days. The pilgrims are distributed in campaigns across Mina according to their geographical origin (i.e., country of origin). The wastewater is disposed of through septic tanks (onsite sewage facilities) that are associated with the pilgrims’ campaign. We conducted the first study to use shotgun-based metagenomic analysis to characterise the abundance and distribution of microbial communities and resistance determinants in wastewater samples from septic tanks in Mina representing different campaigns (European, Middle East and North African (MENA) and East and Southeast Asian countries). The results indicated that high levels of ARGs, including ESBL and aminoglycoside markers, were detected in all sites tested. However, significant variations in the distribution of the bacterial species and the abundance of ARGs were identified. 

Similarly, Kumbh Mela in India represents the world’s largest periodic mass gathering event that involving bathing in small-specified rivers sites. Recent studies have highlighted the striking impact of mass bathing on river ecosystems, including the AMR microbial contents and dissemination of human infectious agents [105,116,117]. A recent study found a nearly 130-fold increase in bacterial load of human origin during the event. Moreover, metagenomic analyses demonstrated an increase in virulence and ARG loads during the MGEs [118].

Many studies have highlighted the roles of surface fresh and aquatic water, rural groundwater and sewage in the dissemination of AMR pathogens. The emergence of AMRs is part of a complicated ecological and evolutionary network, with the use of antimicrobial drugs anywhere within the system potentially selecting for resistance to that drug elsewhere in the network [23]. Gram-negative bacterial resistance, in particular, is promoted through horizontal gene transfer by the acquisition of mobile elements [119,120,121]. There is also increasing evidence that ARGs found in human microbial communities are likely to have been acquired from an environmental source [122,123]. The processing of human, farm and industrial waste together has a significant impact on the emergence of AMR to a wide range of the most clinically effective antibiotics [124,125]. In addition, even treated sewage samples discharged into rivers or lakes from treatment plants may contain significant concentrations of ARGs that enhance the development of AMR bacteria and raise major public health concerns [24,126,127,128].

## Figures and Tables

**Table 1:** Overview of the studies included in this systematic review. AMR: antimicrobial resistant, ARG: antimicrobial resistant genes, ARB: antimicrobial resistant bacteria and MGE: mobile genetic elements.

No.	References	Source of Samples	Types of Investigated Samples	Key Findings
1	**Nahar et al., 2019 [** 35 **] **	Sewage and environmental water	Sewage, river, pond and swimming pool water	AMR bacteria of *E. coli* and *Salmonella* spp. were detected in all environmental samples.
2	**Qin et al., 2019 [** 36 **]**	Sewage	Hospital sewage	Novel species (*Acinetobacter cumulans*) containing ARGs conferring resistance to carbapenems, cephalosporin or aminoglycoside were identified.
3	**Haberecht et al., 2019 [** **37** **] **	Sewage and environmental waters	Sewage water, wastewater treatment plant (WWTP) (influent and effluent) and surface water (ambient water)	Increased abundance of ARB and multidrug resistant (MDR) strains were detected in influent compared to effluent wastewater. Extended-spectrum β-lactamases (ESBL)-producing *E. coli* strains have been identified in environmental surface water.
4	**Sekizuka et al., 2018 [** **38** **]**	Sewage	WWTP effluent	Carbapenem-producing strain of *K. pneumonia* carrying *bla*_KPC-2_ was detected. This novel resistant strain rarely detected in clinical settings in Japan.
5	**Cahill et al., 2019 [** 39 **]**	Sewage and environmental wastewater	Hospital and municipal wastewater (pre- and post-hospital)	Higher rates of carbapenemase-producing *Enterobacteriaceae* (CPE) have been detected in hospital effluent.
6	**Niestepski et al., 2019 [** 40 **]**	Sewage, environmental water and human faeces	Hospital wastewater, WWTP (influent and effluent) and human faeces	The highest drug-resistance levels were observed in the strains isolated from influent and effluent WWTP water. Bacteria of *Bacteroides fragilis* group (BFG) isolated from the WWTPs characterised by higher resistant profiles than those that have been recovered from human and rat faeces.
7	**Hendrieksen et al., 2019 [** 41 **] **	Sewage	Domestic sewage	Clinically relevant ARGs associated with resistance to macrolides, tetracycline, aminoglycoside, beta-lactams and sulfonamides were identified. The abundance of ARGs were higher in samples collected from low-income compared to high-income countries.
8	**Khan et al., 2019 [** **42** **]**	Sewage and environmental waters	Hospital wastewater, WWTP samples and downstream water	β-lactamase genes, including *bla*_IMP−1_, *bla*_IMP−2_ and *bla*_OXA−23_, were detected only in hospital sewage, while *bla*_OXA−48_, *bla*_CTX−M−8_ and *bla*_SFC−1_, *bla*_VIM−1_ and *bla*_VIM−13_ were only detected in downstream river water but not in the WWTP.
9	**Tokajian et al., 2018 [** **43** **]**	Sewage and environmental waters	Refugee camp sewage water and rivers effluent	Higher rates of AMR *E. coli* isolates, including ESBL-producing strains, and those which showed resistance to different antimicrobial drugs, including aminoglycosides, fluoroquinolones and trimethoprim/sulfamethosazole, were detected in samples from refugee camps.
10	**Parnanen et al., 2019 [** **44** **]**	Sewage	Influent and effluent WWTPs samples from different countries	Significantly higher rates of ARGs were identified in effluent samples from low-income compared to high-income countries.
11	**Bougnom et al., 2019 [** **45** **]**	Sewage	Urban wastewater for agriculture (three canals with different settings)	Higher rates of ARG that confer resistance to 11 major antimicrobial drug groups, including aminoglycoside, tetracycline, beta-lactams and macrolides, were detected in urban wastewater. There was difference in the composition of ARGs associated with ESBL within city water from three canals that received water from different environments, including hospitals.
12	**Gouliouris et al., 2019 [** 46 **]**	Sewage	Municipal wastewater (untreated and treated) and hospital sewage	Higher rates of vancomycin and ampicillin-resistant *E. faecium* closely related to hospital isolates have been detected in untreated wastewater plants receiving directly from hospital sewage.
13	**Iweriebor et al., 2015 [** **47** **]**	Sewage	Municipal and hospital wastewater	Ninety-one percent and 100% of the *Enterococcus* spp. (*E. faecalis* and *E. durans*) isolated from the hospital wastewater and final effluent wastewater, respectively, were resistant to vancomycin and erythromycin.
14	**Guo et al., 2017 [** **48** **] **	Sewage	Aerobic-activated sludge (AAS) and an aerobically digested sludge (ADS)	Although MGEs, including plasmids; transposons; integrons (e.g., *intI1*) and insertion sequences (e.g., *ISSsp4*, *ISMsa21* and *ISMba16*) were abundant in both the activated and digested sludge. However, distinct microbial populations were associated with the two sledge samples.
15	**Wang JL, et al., 2015 [** **49** **]**	Sewage	Pharmaceutical WTP (all stages of processing)	The abundance of clinically relevant ARGs, including *sul1*, *sul2*, *tetO*, *tetT*, *tetW* and *tetM*, remained consistently higher throughout the processing stages and discharged into the environment.
16	**Conte D et al., 2017 [** 50 **] **	Sewage and environmental waters	Hospital effluent, sanitary effluent, different sites within WWTP and upstream and downstream river water	ESBL-producing *K. pneumonia* and *E. coli* isolates were higher in hospital effluent and WWTP and river samples, respectively. Quinolone-resistant isolates were identified in hospital effluent, sanitary effluent, outflow sewage and surface water samples. MDR bacteria were detected in the hospital effluent and river waters.
17	**Baumlisberger et al., 2015 [** **51** **]**	Sewage	Up- and downstream wastewater from nursing home	No obvious difference in ARG and MGE abundances were detected between up- and downstream samples.
18	**Adefisoye et al., 2016 [** **52** **]**	Sewage	Final effluents of WWTP	MDR *E coli* isolates associated with neonatal meningitis; intestinal (enterotoxigenic *E. coli* (ETEC), enteropathogenic *E. coli* (EPEC) and enteroaggregative *E. coli* (EAEC)) and ex-intestinal (UPEC) were identified.
19	**Suzuki et al., 2015 [** 53 **] **	Sewage and environmental waters	Effluents of WWTP and surface water	High levels of ARGs associated with resistance to sulfamethosazole and oxytetracycline were detected in environmental surface water.
20	**Froes AM et al., 2016 [** 54 **]**	Sewage	Hospital’s wastewater	Diverse ARGs of serine β-lactamases, including uncommon β lactamase genes *bla*_PER*,*_ *bla*_VEB_ and *bla*_GES,_ were detected in hospital’s wastewater.
21	**Laht M et al., 2014 [** 55 **]**	Sewage	WWTP	High levels of ARGs associated with resistance to tetracycline, sulfonamide and β-lactam were detected in all stages in WWTP wastewater. No difference in ARGs abundance was identified after the purification process.
22	**Walsh et al., 2011 [** **56** **] **	Sewage and environmental water	Seepage water, public tap water and control samples: sewage effluent samples from Wales	*bla*_NDM-1_-producing bacteria were isolated from 17% (12 out 171) and 4% (2 out 50) of seepage and tap water samples, respectively. The detected strains included 11 species in which *bla*_NDM-1_ had not previously been reported (e.g., *Shigella boydii* and *Vibrio cholerae*).
23	**Zhang T et al., 2011 [** **57** **]**	Sewage	Activated sludge of WWTP	Novel plasmids carrying ARGs associated with tetracycline, aminoglycoside and β-lactam resistance were identified. ARGs associated with resistance to tetracycline, macrolide and MDR were highly enriched in the activated sludge.
24	**Chagas TP et al., 2011 [** 58 **] **	Sewage	Influent, clarifier tank effluent and chlorine tank effluent from hospital STP (sewage treatment plant)	Multiresistant and ESBL-producing bacteria (high resistant rates to amikacin, trimethoprim/sulphametoxazole and cefalothin) were identified in the chlorine contact tank effluent.
25	**Szczepanowski et al., 2009 [** **59** **]**	Sewage	Activated sludge samples and final effluent of WWTP	Clinically relevant ARGs associated with resistance to several antimicrobial drugs, including aminoglycoside and β-lactam, were identified in activated sludge and effluent wastewater.
26	**Li D et al., 2010 [** **60** **] **	Sewage and environmental waste water	Wastewater, river water-up (RWU) and -downstream (RWD) associated with oxytetracycline production WWTP	High concentrations of oxytetracycline were identified in wastewater and in river water downstream but not in upstream waters. MDR phenotypes isolates were identified in the WW and RWD and less frequent in RWU.
27	**Zhang H et al., 2019 [** **61** **]**	Sewage	Samples from 18 WWTPs	Activated sludge was the main reservoir of ARGs associated with resistance to sulfonamide (*sul1* and *sul2*) and tetracycline (*tetW, tetX* and *tetQ*).
28	**Li, B et al., 2015 [** **62** **]**	Sewage, environmental water and faecal samples	Samples from AAS and ADS and different environmental waters and faecal samples (human, chicken and pig)	High level of ARGs, including those associated with MDR, and resistance to bacitracin; tetracycline; β-lactam; macrolide, lincosamide and streptogramin (MLS); aminoglycoside; quinolone and sulphonamide were detected in all samples.
29	**Hembach et al., 2017 [** **63** **]**	Sewage	Influent and effluent water from seven WWTPs	*mcr-1* (associated with resistance to colistin) was detected in influent samples of all seven WWTPs and some of effluent waters (i.e., it was not eliminated during wastewater treatment reaching the aquatic environment). AMR strains of *A. baumannii*, *E. coli* and *K. pneumonia* were detected in both influent and effluents samples.
30	**Igbinosa IH et al., 2012 [** **64** **]**	Sewage	WWTP	AMR *Aeromonas* spp. isolates resistant to penicillin, oxacillin, ampicillin and vancomycin were identified. Class A pse1 β-lactamase, class 1 integron and the *bla*_TEM_ gene were detected in 20.8%, 20.8% and 8.3% of the identified isolates, respectively.
31	**Igbinosa EO et al., 2011 [** **65** **]**	Sewage	Final effluents WWTP	*Vibrio* spp. strains (including *V. parahaemolyticus*, *V. fluvialis* and *V. vulnificus*) resistant to erythromycin, chloramphenicol, nitrofurantoin, cefuroxime and cephalothin were detected. SXT antibiotic resistance gene cluster (*floR*, *strB*, *sul2*, *dfrA18* and *dfrA1*) were also identified in % of these strains.
32	**Johnning A, et. Al 2015 [** **66** **] **	Sewage and environmental water	Up and downstream WWTP and from industrially polluted sites and sediment samples from a pristine lake	Mutations in chromosomal genes *gyrA* and *parC*, associated with resistance to fluoroquinolone, were detected in *E. coli* communities. High abundance of mutations was correlated with the concentration of fluoroquinolones in investigated samples (i.e., samples polluted with high concentrations of fluoroquinolone).
33	**Sahlström L et al., 2009 [** **67** **] **	Sewage and clinical samples	WWTP and isolates from humans and chickens	Vancomycin-resistant isolates of *Enterococcus* spp., including *E. faecium*, *E. hirae* and *E. durans,* were detected.
34	**Araújo C et al., 2010 [** **68** **]**	Sewage	Sludge and sewage of urban and poultry slaughter house WWTP	Vancomycin-resistant isolates of *Enterococcus* spp., including *E. faecium*, *E. gallinarum* and *E. casseliflavus*, which were also resistant to varied groups of antimicrobial drugs (kanamycin, tetracycline, erythromycin, ciprofloxacin, ampicillin, streptomycin and gentamicin), were detected.
35	**Soge O et al., 2009 [** **69** **] **	Sewage and environmental water	Water, soil and sewage	The majority of *Clostridium perfringens* strains recovered from water samples were found to carry more than one ARG encoding resistance to tetracycline and erythromycin.
36	**Zhang X et al., 2009 [** **70** **]**	Sewage	WWTP	Enterobacteriaceae strains carrying class 1 integrons and ARGs associated with resistance to trimethoprim (*dfr17*) and streptomycin (*aadA5*) were detected.
37	**Odjadjare EO et al., 2010 [** **71** **]**	Sewage	WWTP final effluent, discharge point and upstream and downstream of the discharge point	Most of the *Listeria* spp. isolates recovered from final effluents were MDR strains.
38	**Okoh AI et al., 2010 [** **72** **] **	Sewage	WWTP final effluents	MDR *Vibrio* spp. strains that showed resistance to varied antimicrobial drugs (including sulfamethoxazole, trimethoprim, cotrimoxazole, chloramphenicol, streptomycin, ampicillin, tetracycline, nalidixic acid and gentamicin) were detected.
39	**Yang H et al., 2010 [** **73** **]**	Sewage and environmental waters	Soil, water and faecal samples	Higher copies of tetracycline ARGs and higher abundance of tetracycline-resistant bacteria were identified in farm (cattle), compared to nonfarm, wastewater samples.
40	**Garcia-Armisen T et al., 2011 [** **74** **]**	Environmental water	Sewage-contaminated rivers	Most of the ARB detected in the Zenne river, downstream of Brussels, were MDR strains. The abundance of AMR communities (heterotrophic and faecal bacteria) was not correlated with the level of contamination of river water with sewage.
41	**Colomer-Lluch M et al., 2011 [** **75** **]**	Sewage and environmental water	Urban sewage and river water	β-lactamase genes (*bla*_TEM_ and *bla*_CTX-M9_) and one encoding penicillin-binding protein (*mecA*) were detected in the DNA phages recovered from all the samples.
42	**Fuentefria DB et al., 2011 [** **76** **]**	Sewage and environmental water	Hospital wastewater and superficial water	Genetically distinct populations of AMR *Pseudomonas aeruginosa* were detected in these different environments (hospital wastewater and superficial water that received this wastewater discharge).
43	**Gaze WH et al., 2011 [** **77** **]**	Sewage	Industrial waste, sewage sludge and pig slurry	Higher prevalence of class 1 integrons was detected in bacteria recovered from sewage sludge and pig slurry (exposed to antibiotic residues and detergents) compared to agricultural soils to which these waste products are amended. It has been estimated that ~10^19^ bacteria carrying class 1 integrons enter the United Kingdom environment by disposal of sewage sludge each year.
44	**Ma L et al., 2011 [** **78** **] **	Sewage	WWTP	The abundance of bacteria (*E. coli*, *Klebsiella* spp. and *Aeromonas veronii*) carrying class I integronase gene *intI1* were higher in effluent compared to influent wastewater. *intI1* was detected in 20.4%, 30.9% and 38.9% of bacteria recovered from influent, activated sludge and effluent wastewater, respectively. This study suggested a role of activated sludge (characterized by high biomass and biodiversity) in developing AMR through the dissemination of integrons.
45	**Mokracka J et al., 2011 [** **79** **]**	Sewage	WWTP	Quinolone- and fluoroquinolone-resistant strains constituted 56% and 50.4% of recovered integron-bearing *E. coli* strains (including diarrheagenic and extraintestinal strains carrying virulence traits), respectively. Virulent extraintestinal strains constituted ~50% of all isolates and were detected in samples recovered from all wastewater treatment stages, including final effluent.
46	**Amaya E et al., 2012 [** **80** **] **	Sewage and environmental waste water	Hospital wastewater and well waters	High levels of MDR *E. coli* isolates were recovered from samples collected from both hospital wastewaters and environmental well water. *E. coli* strains harbouring *bla*_CTX-M1_ and *bla*_CTX-M9_ were predominated in samples collected from wells and hospital wastewater, respectively.
47	**Mokracka J et al., 2012 [** **81** **] **	Sewage	Municipal WWTP	MDR *Enterobacteriaceae* strains carrying class 1 and class 2 integrons (12.1%; 221 out of 1832) were identified in different stages of a municipal wastewater treatment plant (61.5%, 12.7% and 25.8% of ARB were originated from raw sewage, aeration tank and final effluent, respectively). The abundance of ARGs and MDR bacteria, particularly the level of ARG diversity and B-lactamase-producers, were higher in final effluent samples.
48	**Splindler A et al., 2012 [** **82** **]**	Sewage	Untreated hospital effluents	Half of *Pseudomonas* spp. isolates recovered from untreated hospital effluent wastewater were MDR strains, while 41.9% (52 out of 124) of the isolates were found to carry *intlI*.
49	**Gundogdu, A. et al., 2012 [** **83** **] **	Sewage	Untreated hospital wastewaters and WWTP	High level of ESBL-producing *E. coli* isolates were detected in untreated hospital wastewaters (*bla*_SHV_), with distinct genotypes (*bla*_CTX-M_) associated with the samples recovered from WWTP.
50	**Zarfel, G et al., 2013 [** **84** **]**	Sewage and clinical samples	Sewage and human urinary tract infection samples	ESBL-producing bacteria carrying *bla*_CTX-M_ were predominated in both sewage sludge (*bla*_CTX-M-15_) and UTI (*bla*_CTX-M-1_) samples. The study suggested the occurrence of a genetic exchange between the ESBL-resistant *E. coli* populations from human infections and those present in sewage sludge.
51	**Colomer-Lluch M et al., 2013 [** **85** **]**	Sewage and environmental water	Sewage and river water samples	Quinolone-resistant *E. coli* strains of clinically relevant ST69 and ST131 (carrying virulence traits) predominated in samples recovered from urban wastewater and both river and wastewaters, respectively. Similar virulence and macro-restriction profiles were identified in environmental and human isolates of ST131.
52	**Sadowy E et al., 2014 [** **86** **]**	Sewage and environmental water	Wastewater, riverine estuary and anthropogenically impacted marine catchment basin	AMR isolates of *Enterococcus* spp., especially fluoroquinolone- and aminoglycoside-resistant *E. faecium* that shared virulence determinants and ST similar to nosocomial high-risk enterococcal clonal complexes (HiRECC), were detected.
53	**Gao P et al., 2015 [** **87** **]**	Sewage	WWTP	Positive correlations were observed between the occurrence of heavy metals (e.g., zinc and lead and *ereB*, *mefA*&*E* and *ermB*) and antibacterial residues (e.g., triclosan with *ereA*, *ereB*, *mefA*&*E* and *ermB*) in urban wastewaters and the abundance of erythromycin-resistant genes.
54	**Nishiyama M et al., 2015 [** **88** **]**	Sewage and environmental water	Sewage and urban river water samples	vanC-type vancomycin-resistant *E. faecium* and *E. faecalis*, which are the major types of enterococci in humans, were detected in both sewage and urban river water samples.
55	**Zhang S et al., 2015 [** **89** **]**	Sewage	WWTP	Gram-negative and -positive isolates dominated WWTP influent and effluent samples, respectively. The frequency of detection of tetracycline-, sulphonamide-, streptomycin- and β-lactam-resistance genes (except *sulA* and *bla*_CTX-M_) were higher in ARB from influent compared to effluent samples. The abundances of ARGs in activated sludge were higher in aerobic compartments than in anoxic ones.
56	**Simo Tchuinte PL et al., [** **90** **]**	Sewage	Hospital effluent and sludge	Novel class 3 integrons with oxacillinase gene cassette, including aminoglycoside and β-lactam-resistant genes (*bla*_OXA-10_, *bla*_OXA-368_ or *bla*_OXA-2_), were identified in *Acinetobacter johnsonii*, *Aeromonas allosaccharophila* and *Citrobacter freundii,* which were recovered from hospital effluent samples.
57	**Young S et al., 2016 [** **91** **]**	Sewage and environment water	Water and sediment samples from sewage spill site	Nosocomial pathogen; vancomycin-resistant *E. faecium* (harbouring *vanA* associated with a high resistance level) were isolated from water and sediment for up to 3 days after a sewage spill. *vanA* gene were found to persist for an additional week within these environments. Culturable levels of enterococci in water exceeded recreational water guidelines for 2 weeks following the spill, declining about five orders of magnitude in sediments and two orders of magnitude in the water column over 6 weeks.
58	**Lee J et al., 2017 [** **92** **]**	Sewage	Food waste-recycling wastewater (FRW), manure and sewage sludge	The abundance of ARGs was greatest in manure, followed by sewage sludge and FRW. However, different patterns in the diversity and mechanisms of ARGs were identified. ARG associated with β-lactam resistance were higher in the FRW, and sulfonamides-resistant genes are higher in sludge. Total ARGs is associated with class 1 integron only in manure and sludge.
59	**An XL et al., 2018 [** **93** **]**	Sewage	Influent, activated sludge and effluents of urban WWTP	High concentration of class 1 integron gene cassette (including trimethoprim, aminoglycoside and beta-lactam resistance genes) were identified in activated sludge.
60	**Haller L et al., 2018 [** **94** **]**	Sewage	Hospital effluents	MDR bacteria belonging to *Enterobacteriaceae* and other species, including ESBL- and carbapenemase-producers, were identified.
61	**Galler H et al.,2018 [** **95** **]**	Sewage	Activated sludge	Clinically relevant ARBs, including ESBL-*Enterobacteriaceae*, MRSA and vancomycin-resistant *Enterococcus* spp., were detected. ARG associated with resistance to β-lactam, vancomycin (*vanA*) and methicilin (*mecA*) were identified.
62	**Quach-Cu J et al., 2018 [** **96** **]**	Sewage	Raw wastewater, activated sludge and secondary and tertiary WWTP effluent	The abundance of *bla*_SHV_, *bla*_TEM_ and *sul1* were higher in raw wastewater than other samples.
63	**Yousfi K et al., 2019 [** **97** **]**	Sewage	Hospital effluents	*Enterobacteriaceae* isolates (including *E. coli* and *K. pneumoniae*) and non-*Enterobacteriaceae* Gram-negative bacterial isolates (including *A. baumannii* and *A. hydrophila*) showed high levels of resistance to β-lactam and non-β-lactam-antibiotics, and most of them are multidrug-resistant. This study is the first study that found genes encoding carbapenemases, including *bla*_OXA-23_ and *bla*_OXA-48_, like in *A. baumannii*, *K. oxytoca* and *S. xiamenensis* in Algerian hospital effluents.

## Supplementary Materials

The following are available online at https://www.mdpi.com/2414-6366/5/1/33/s1, Table S1: Characterisation of studies detecting disseminated AMR genes in sewage.
