# Supplementary material for: The Contribution of Wastewater to the Transmission of Antimicrobial Resistance in the Environment: Implications of Mass Gathering Settings
Source: Trop Med Infect Dis. 2020 Feb 25;5(1):33. doi: 10.3390/tropicalmed5010033 (PMC7157536; doi:10.3390/tropicalmed5010033)
Supplement: Supplementary file 1 [file tropicalmed-05-00033-s001.pdf]

Table S1: Characterisation of studies detecting disseminated AMR genes in sewage

Country/ study site	Year of sample collection	Targeted/detected ARB	ARG associated with resistance to	AMR Molecular Detection		Phenotypic/ genotypic analysis	Reference
				Technology	Platform		
Bangladesh/ Mymensing	2018	*E. coli* and *Salmonella* Spp.	Tetracycline (*tetA*).	PCR	-	Both	[35]
China/<br><br>West China	2017-2018	*Acinetobacter cumulans* (novel species)	Carbapenems (*bla*$_{\text{NDM-1}}$, *bla*$_{\text{OXA-58}}$ and *bla*$_{\text{OXA-23}}$); other β-lactams (*bla*$_{\text{PER-1}}$); aminoglycosides (*aac(6′)-Ib*, *aac(3)-IId* and *aph6)*; bleomycin (*ble)*; macrolides (*mphE* and *msrE*); rifampin (*arr-3*); sulfonamides (*sul1* and *sul2)* and tetracycline (*tet39* and *tetY*).	WGS	Illumina HiSeq	Both	[36]
USA/<br>Larimer County, Colorado	2016-2017	*E. coli*	Carbapenems (e.g., *bla*$_{\text{OXA}}$, *bla*$_{\text{CTX-M}}$, and *bla*$_{\text{TEM}}$); aminoglycoside (e.g., *aac(6′)*, *aac(3)* and *aph(6))*; fluoroquinolone (*qnr* and *mfd);* phenicol (*cat*) and sulfonamid (*sul*).	PCR and WGS	Illumina MiSeq	Both	[37]
Japan/	2017	*K. pneumoniae*	Carbapenems (*bla*$_{\text{KPC-2}}$).	WGS	Illumina N	Both	[38]

Tokyo Bay					extSeq		
Ireland *	2017	*Enterobacteriaceae (E. coli, Enterobacter cloacae* complex, *K. pneumoniae, Citrobacter freundii* and *Klebsiella oxytoca)*	Carbapenems *(bla*$_{NDM}$, *bla*$_{OXA-48}$, *bla*$_{IMP}$ and *bla*$_{VIM}$).	PCR and WGS	Illumina HiSeq	Both	[39]
Poland/ Olsztyn	2016	*Bacteroides fragilis* group (*Parabacteroides distasonis, B. fragilis, B. thetaiotaomicron, B. ovatus* and *B. cacae*)	Fluoroquinolone (*bexA, qnrB* and *qnrS*); macrolide; lincosamide and streptogramin (*ermF, linA* and *mefA*); tetracycline (*tetQ, tetA, tetB, tetL* and *tetM*); chloramphenicol (*catA1*) and β-lactam (*cepA* and *cfxA*).	PCR	-	Both	[40]
Global collection from 60 countries (74 cities)	2016	ND[#]	β-lactam (e.g., variants of *bla*$_{OXA}$); aminoglycoside (*aadA);* tetracycline (e.g., *tetQ* and *tetW*); sulfonamide (variants of *sul1*); macrolide (e.g., *mphE, msrD, msrE, ermB, ermF* and *mefA*) and streptomycin (e.g., *strA* and *strB*).	WGS	Illumina HiSeq	Genotype	[41]

Sweden/<br>Örebro	2016	ND[#]	β-lactam (e.g., *bla*$_{\text{CTX-M-1}}$, *bla*$_{\text{SHV-156G}}$, *bla*$_{\text{SHV-238G240E}}$, *bla*$_{\text{ACT-1}}$, *bla*$_{\text{ACT-5/7}}$, *bla*$_{\text{CMY-10}}$, *bla*$_{\text{DHA}}$, *bla*$_{\text{FOX}}$, *bla*$_{\text{LAT}}$, *bla*$_{\text{MIR}}$ and *bla*$_{\text{MOX}}$); fluoroquinolone (variants of *qnrB-1* and *aacC2*); macrolide (*mefA*); aminoglycoside *(aadA1)*; tetracycline (*tetA* and *tetB*) and macrolide-lincosamide-streptogramin B (*ermB*).	qPCR	-	Genotype	[42]
Lebanon/<br>Al-Qaa refugee camp	2016	*E. coli*	β-lactam *(bla*$_{\text{CTX-M-14}}$, *bla*$_{\text{OXA-1}}$, *bla*$_{\text{SHV-12}}$ and *bla*$_{\text{CMY-2}}$); aminoglycoside *(aac(6)-Ib, acc(3)-II* and *acc(3)-II);* fluoroquinolone *(*variants of *gyrA* and *parC)* and *int-I1*.	PCR and DNA sequencing	Sanger sequencing	Both	[43]
Multiple countries**	2015-2016	ND[#]	Aminoglycoside *(aadA* and *strB*); β-lactam (e.g., *bla*$_{\text{OXA}}$, *bla*$_{\text{GES}}$ and *bla*$_{\text{VEB}}$); macrolide–lincosamide–streptogramin B (*ereA*, *ermF,* and *matA*/*mel*); sulfonamides (*sul1*); tetracyclines (*tetM* and *tetQ*); amphenicol (*cmxA*) and quinolone (*qnrSrtF11*).	PCR	-	Genotype	[44]
Burkina Faso/<br>Ouagadougou	2015	ND[#]	Sulfonamide (including *sul1* and *sul2*); aminoglycoside (e.g., *aadA13*, *aadA* and *aadA15*); tetracycline (*tetB*); β-lactam (e.g., *bla*$_{\text{OXA-226}}$, *bla*$_{\text{OXA-256}}$, *bla*$_{\text{OXA-347}}$, *bla*$_{\text{OXA-46}}$, *bla*$_{\text{SHV-100}}$, *bla*$_{\text{GES-21}}$ and *bla*$_{\text{AIM-1}}$) and macrolide-lincosamide-streptogramin B (e.g., *ermB* and *ermF*).	WGS	Illumina HiSeq	Genotype	[45]

United Kingdom/ East of England	2014-2015	*Enterococcus faecium*	Vancomycin (*vanA)*; aminoglycoside (*aac(6′)-aph(2″)* and *aacA*); trimethoprim (*dfrG)*; macrolide-lincosamide-streptogramin B (*msrC, ermB* and *ermF*); spectinomycin (*spw*); chloramphenicol (*cat*) and tetracycline (*tetM* and *tetS*).	DNA sequencing	Illumina HiSeq	Both	[46]
South Africa/ Alice	2014	*Enterococcus spp.*	Vancomycin (*vanB, vanC1* and *vanC2/3*) and macrolide (*ermB*).	PCR	-	Both	[47]
China/ Beijing	2013	ND#	Sulfonamide (e.g., *sul1, sul2* and *sul 3*); aminoglycoside (e.g., *aac3* and *aac6*); tetracycline (e.g., *tet41* and *tetC*); β-lactam (e.g., bla_{penA} and bla_{pepEC}); macrolide-lincosamide-streptogramin B (e.g., *msrE* and *ermF*); fluoroquinolone (*oqxBgb*); vancomycin (*vanR-F* and *van R-M*) and trimetophrim (*dfrK*).	WGS	Illumina HiSeq	Genotype	[48]
China/ Northern China	2013	ND#	Tetracycline (*tetO, tetT, tetQ, tetW* and *tetM*); β-lactam *(*bla_{OXA-1}); sulphonamide (*sul1* and *sul2*) and macrolide (*ermB*).	PCR	-	Genotype	[49]

Brazil/<br>Curitiba	2012-2013	*E. coli, K. pneumoniae* and *K. oxytoca*	β-lactam (e.g., *bla*$_{CTX-M}$, *bla*$_{SHV}$ and *bla*$_{GES-5}$) and fluoroquinolone (*oqxAB, aac-6'-Ib-cr, qnrS, qnrB* and variants of *gyrA*).	PCR and DNA sequencing	Sanger sequencing	Both	[50]
Germany/<br>South-west Germany	2012-2013	ND#	Aminoglycoside (*ant(3")*); β-lactam (*bla*$_{OXA}$); macrolide (*ermB* and *macB*); multidrug (*acrB*); quinolone (*parC* and *gyrA*); rifamycin (*rpoB*) and tetracycline (*tetW, tetO* and *tet32*).	WGS	Illumina HiSeq	Genotype	[51]
South Africa/<br>Amathole District Municipality	2012-2013	*E. coli*	Aminoglycosides (*strA* and *aadA*); phenicols (*catI, catII* and *cmlA1*); β-lactam (*bla*$_{TEM}$ and *ampC*) and tetracyclin (*tetA, tetB, tetD, tetK* and *tetM*).	PCR	-	Both	[52]
South Africa<br>The kwini	2012	Not specify**	Sulfonamide (*sul1, sul2* and *sul3*) and tetracycline (*tetM*).	PCR and DNA sequencing	Sanger sequencing	Both	[53]
Brazil/<br>Rio de Janeiro	2011	ND#	β-lactam (*bla*$_{CfxA}$, *bla*$_{ACC}$, *bla*$_{OXA-10}$, *bla*$_{CEPA}$ and *bla*$_{Fox}$).	WGS	Roche 454 sequencer	Genotype	[54]
Finland/<br>Helsinki<br>Estonia/<br>Tartu and Tallinn	2010-2011	ND#	Tetracycline (*tetC* and *tetM*); β-lactam (*bla*$_{oxa-58}$, *bla*$_{shv-34}$ and *bla*$_{ctx-m-32}$) and sulphonamide (*sul1* and *sul2*).	PCR	-	Genotype	[55]

India/ New Delhi	2010-2011	*V. cholerae, E. coli, Aeromonas caviae, Shigella boydii, Enterobacteria* and *Aeromonas*	β-lactam (*bla*NDM-1).	PCR	-	Both	[56]
China/Hong Kong, Shatin	2010	ND#	β-lactam (*ampC, bla*VEB-3, *bla*VIM-2 and *bla*IMP-1); aminoglycoside (*aacA4, aadA1, aadA2, aadA2b* and *aadA24*); sulphonamides (*sulI*); trimethoprim (*dfrA1*); tetracycline (*tetA, tetC, tetG, tetM* and *tetB*); macrolide (*ermB* and *macB*) and aminoglycoside (*aadB*).	PCR and WGS	Illumina HiSeq	Genotype	[57]
Brazil/ Rio de Janeiro	2008	*K. pneumoniae, Enterobacter cloacae* and *E. coli*	β-lactam (*bla*TEM, *bla*SHV and *bla*CTX-M).	PCR and DNA sequencing	Sanger sequencing	Both	[58]
Germany/ Bielefeld-Heepen	2006	Not specified**	Aminoglycoside (e.g., *aacA, aac(6')* and *aph*); β-lactam (e.g., *bla*TLA-2, *bla*CTXM-27, *bla*GES-3, *bla*IMP-13 and *bla*OXA-58); chloramphenicol (e.g., *cmxA* and *cat*); fluoroquinolone (e.g., *qnr* and *qnrB4*); erythromycin (*ereA2* and *arr2*); tetracycline (*tetB(P), tetL, tetM, tetS* and *tetX*); sulfonamide (*sul1* and *sul3*); trimethoprim (*dfrD*) and multidrug (*mexD, mexI* and *mexY*).	PCR and DNA sequencing	Sanger sequencing	Both	[59]
China/ North China	2004-2005	Multiple bacteria***	Tetracycline (*tetA, tetW, tetC, tetJ, tetL, tetD, tetY* and *tetK*).	PCR and DNA sequencing	Sanger sequencing	Both	[60]

China/ Southern, Northern and Eastern regions	‡	ND[#]	Sulphonamide (*sul1* and *sul2*); tetracycline (*tetX, tetW* and *tetQ*) and class 1 integron (*intl1*).	PCR and WGS	Illumina MiSeq	Genotype	[61]
China/<br><br>Hong Kong	‡	ND[#]	aminoglycoside (e.g., *aadA*); bacitracin (*bacA*); macrolide-lincosamide-streptogramin B (e.g., *macB, mefA* and *ermB*); tetracycline (*tet32, tetM, tetQ* and *tetO*); sulfonamide (*sul1,2*); vancomycin (*vanR*); trimetophrim (*dfrA*); quinolone (*qnrS*); β-lactam (e.g., bla_{OXA-1}, bla_{TEM}, bla_{CMY-2} and bla_{CTX-M}) and multiple drugs (e.g., *acrA, mdtH, mdtL* and *mdtO*).	WGS	Illumina HiSeq	Genotype	[62]
Germany*	‡	*E. coli, K. pneumoniae* and *A. baumannii*	Colistin (*mcr-1*); erythromycin (*ermB*); tetracycline (*tetM*); β-lactam (e.g., bla_{OXA1}, bla_{TEM}, bla_{CMY-2} and bla_{CTX-M}) and chloramphenicol (*catA2* and *cmlA5*).	PCR	-	Genotype	[63]

South Africa/ Fort Beaufort and Alice	‡	*Aeromonas* spp.	β-lactam (*bla*TEM) and class 1 integron.	PCR	-	Both	[64]
South Africa/ Fort Beaufort and Alice	‡	*Vibrio* spp.	Dulfonamide (*sul2*), chloramphenicol (*floR*), trimetophrim (*dfr18* and *dfrA1*), tetracycline (*tetA*), streptomycin (*strB*) and β-lactam (*bla*TEM).	PCR	-	Both	[65]
India/ Hyderabad Sweden / Skövde	‡	*E. coli*	Quinolone (variants of *GyrA* and *ParC*).	PCR and WGS	the GS FLX+ system	Genotype	[66]
Swedish/ Uppsala	2003	*Enterococcus hirae, E. faecium* and *E. durans*	Vancomycin (*vanA* and *vanB*).	PCR	-	Both	[67]
Portugal/ Continental territory	ND	*E. faecium, E. gallinarum* and *E .casseliflavus*	Vancomycin (*vanA* and *vanC1/2*).	PCR	-	Both	[68]
USA****	2003-2006	*Clostiridium perfringens*	Tetracycline (*tetA, tetB* and *tetM*) and macrolide (*ermB, ermQ* and *mefA*).	PCR and DNA sequencing	Sanger sequencing	Genotype	[69]

China/<br>Hong Kong and Shanghai<br><br>USA/Sao Paolo	2007-2008	*Enterobacteriaceae*	Trimetophrim (*dfr17*) and spectinomycin/streptomycin (*aadA5*).	PCR and DNA sequencing	Sanger sequencing	Both	[70]
South Africa/<br><br>Eastern Cape	2007-2008	*Listeria ivanovii* and *L. innocua*	Sulphonamide (*sulII*).	PCR	-	Both	[71]
South Africa/<br><br>Eastern Cape	2010	*Vibrio* spp.	Trimethoprim (*dfr18* and *dfrA1*), chloramphenicol (*floR*), tetracycline (*tetA*), streptomycin (*strB*) and sulfamethoxazole (sulII).	PCR	-	Both	[72]
USA/<br><br>Northern Colorado	2008-2009	ND#	Tetracycline (*tetB, tetC, tetO* and *tetW*).	PCR	-	Both	[73]
France/<br>Seine River<br><br>Belgium/<br>Scheldt River	2008-2009	Heterotrophic bacteria	ND	PCR and DNA sequencing	Sanger sequencing	Both	[74]
Spain/<br><br>Barcelona	‡	MRSA and *E. coli*	β-lactam (*bla*$_{TEM}$ and *bla*$_{CTX-M}$) and penicillin-binding protein (*mecA*).	PCR and DNA sequencing	Sanger sequencing	Genotype	[75]

Brazil/<br>Rio Grande	2005 and 2007	*Pseudomonas aeruginosa*	ND	PCR	-	Both	[76]
United Kingdom	‡	ND#	Class 1 integron.	PCR	-	Genotype	[77]
China/<br>Jiangshou	2010	*E. coli, Klebsiella* spp. and *Aeromonas* spp.	Class 1 integronases (*intI1* and *qacED1*) and sulfonamid (*sulI*).	PCR	-	Genotype	[78]
Poland	2008-2010	*E. coli*	Class 1 and 2 integron.	PCR	-	Genotype	[79]
Nicaragua/<br>Leon	20008-2009	*E. coli*	β-lactam (*bla*CTX-M, *bla*SHV, *bla*OXA and *bla*TEM).	PCR and DNA sequencing	Sanger sequencing	Both	[80]
Poland	2008-2010	*Enterobacteriaceae*	Class 1 and 2 integron.	PCR and DNA sequencing	Sanger sequencing	Both	[81]
Brazil	2007	*Pseudomonas* spp.	Class 1 integron.	PCR	-	Both	[82]
Australia/<br>Queensland	2011	*E. coli*	β-lactam, including *bla*TEM, *bla*SHV, *bla*CTX-M and *bla*OXA.	PCR and DNA sequencing	Sanger sequencing	Both	[83]

Austria/<br>Graz and Styria	2009	*E. coli*	β-lactam (*bla*$_{CTX-M-15}$, *bla*$_{TEM}$ and *bla*$_{SHV}$).	PCR and DNA sequencing	Sanger sequencing	Both	[84]
Spain/<br>Catalonia	2009-2010	*E. coli*	β-lactam (*bla*$_{CTX-M-15}$, *bla*$_{TEM}$ and *bla*$_{SHV}$).	PCR	-	Both	[85]
Poland/<br>Gulf of Gdansk	2011	*Enterococcus* spp.	Tetracyclin (*tetM, tetL* and *tetS*); fluoroquinolon (variants of *gyrA* and *parC*); streptomycin (*ant(6')-Ia*) and glycopeptide (*aac(6')-Ie-aph(2")*).	PCR and DNA sequencing	Sanger sequencing	Both	[86]
China/<br>Shanghai	2013-2014	ND#	Erythromycin (*ereA, ereB, ermA, ermB, ermC, mefA/mefE* and *msrA/msrB*).	PCR	-	Both	[87]
Japan/<br>Miyazaki	‡	*Enterococcus* spp.	Vancomycin (*vanC1* and *vanC2/C3*).	PCR and DNA sequencing	-	Both	[88]
China/Jiangsu	‡	Heterotrophic bacteria	β-lactam (*bla*$_{TEM}$, *bla*$_{SHV}$ and *bla*$_{CTX\_M}$); sulfonamide (*sul1, sul2* and *sul3*); tetracycline (*tetA, tetB, tetE* and *tetO*) and streptomycin (*strA* and *strB*).	PCR	-	Both	[89]
France/<br>Limoges<br>Luxembourg/<br>Esch-sur Alzette	2010-2011	*Acinetobacter johnsoni, Aeromonas allosacharophiles* and *Citrobacter* spp.	Class 3 integron with oxillinase gene cassette.	PCR	-	Both	[90]

United States/Florida	2014	*Enterococcus* spp.	Vancomycin (*vanA*).	PCR and WGS	Illumina	Both	[91]
South Korea/	‡	ND#	Tetracycline (*tetM, tetQ, tetA, tetB, tetE, tetG, tetH* and *tetX*); sulfonamides (*sul1* and *sul2*); macrolides (*ermB* and *ermC*); quinolones (*qnrD*) and β-lactam (bla_{TEM}).	PCR	-	Genotype	[92]
China/Xiangmen and LongYan	2014	ND#	Aminoglycosides, beta-lactams, bicyclomycin, chloramphenicol, fosfomycin, gentamicin and macrolides.	PCR and WGS	Illumina Miseq300	Genotype	[93]
Singapore	2014	*Pseudomonas* spp., *Klebsiella* spp., *Enterobacter* spp. and *Citrobacter* spp.	β-lactam (bla_{SHV}, bla_{NDM-1}, bla_{CTX} and bla_{KPC}).	PCR	-	Both	[94]
Austria/Graz	2011-2012	Enterobacteriaceae, MRSA and vancomycin-resistant *Enterococci*	β-lactam (bla_{CTX-M}).	PCR and DNA sequencing	Sanger sequencing	Both	[95]
China	‡	ND#	$bla_{SHV/TEM}$ and *sul1*.	PCR	-	Genotype	[96]

Algeria/<br><br>Bejaia and Tizi Ouzou	2011- 2012	*E. coli*, *Klebsiella pneumoniae*, *Acinetobacter* spp., *Aeromonas* spp. and *Pseudomonas* spp.	β-lactam (*bla*$_{CTX-M}$, *bla*$_{TEM}$, *bla*$_{SHV}$, *bla*$_{OXA-48-like}$, *bla*$_{OXA-23}$ and *bla*$_{OXA-51}$); quinolone (*qnrB* and *qnrS*); tetracycline (*tetA, tetB* and *tetC*); trimethoprim (*dfrA1*); aminoglycoside (*aac(3)-IIc (aacC2)* and *aac(6')-1b*) and sulfonamid (*sul1* and *sul2*).	PCR	-	Both	[97]

* - study site is not mentioned.

# - identification of bacteria species was not performed.

** - Portugal, Spain, Ireland, Cyprus, Germany, Finland and Norway.

**** - Arizona, California, Florida, Idaho, Indiana, Iowa, Georgia, Maryland, Massachusetts, Montana, North Carolina, Pennsylvania, Texas and Washington States.

‡ - year of collection was not mentioned.
